# Quantitative MRI evaluation of whole abdomen adipose tissue volumes in healthy volunteers—validation of technique and implications for clinical studies

**DOI:** 10.1259/bjr.20180025

**Published:** 2018-03-30

**Authors:** Matthew Marzetti, Tracy Brunton, Laura McCreight, Ewan Pearson, Stephen Docherty, Stephen J Gandy

**Affiliations:** 1 NHS Tayside Medical Physics, Ninewells Hospital, Dundee, UK; 2 NHS Tayside Clinical Radiology, Ninewells Hospital, Dundee, UK; 3 Division of Molecular and Clinical Medicine, University of Dundee, Ninewells Hospital, Dundee, UK

## Abstract

**Objective:**

To explore “whole abdomen” MRI methods for quantifying adipose tissue volumes and to establish associations with body mass index (BMI) and measurement reproducibility—relative to existing “partial abdomen” methods.

**Methods:**

15 healthy volunteers were scanned on a 3T MRI scanner using a double-echo three-point-Dixon gradient echo sequence. Whole abdomen volumes were acquired via three separate scans (“supine 1”, “supine 2” and “prone”). Segmentation was applied to derive (i) “whole abdomen” visceral (VAT) and subcutaneous adipose tissue (SCAT) volumes, and (ii) “partial abdomen” volumes at the lumbar spine (L3 to L5). Root-mean-square coefficients of variation (RMS CoV) were calculated to quantify the variability of each measurement.

**Results:**

“Whole abdomen” measurements were found to correlate better with BMI (r^2^_max_ = 0.74) than “partial abdomen” volumes (r^2^_max_ = 0.66). Total adipose tissue (TAT) measurements correlated better with BMI (r^2^_max_ = 0.74) than SCAT (r^2^_max_ = 0.43) or VAT (r^2^_max_ = 0.33) for both methods. Scan-to-scan RMS CoV’s for “whole abdomen” VAT and SCAT measurements were 4.16 and 3.61% compared to 6.31 and 5.07% for “partial abdomen” measurements.

**Conclusion:**

“Whole abdomen” measures of abdominal adiposity are better correlated with BMI and demonstrate better scan-to-scan reproducibility than “partial abdomen” measures. It is recommended that “whole abdomen” measures be used in longitudinal MRI radiology investigations, where small volume changes may occur.

**Advances in knowledge:**

Whole abdomen adipose tissue volumes can be measured and quantified using commercial MRI sequences and post-processing software. These methods are better correlated with BMI and are more reproducible than partial abdomen measures.

## INTRODUCTION

Human obesity is a major healthcare problem across the developed world, and recent statistics published by the World Health Organisation (WHO) has shown that 64.2% of the UK adult population are estimated to be overweight, with 26.9% estimated to be obese.^1^ The health implications of obesity are widely reported, and can include greater “multifactorial” risks associated with liver diseases (*e.g.* non-alcoholic fatty liver disease—NAFLD),^2^ cancer,^3^ cardiovascular diseases^4^ and diabetes^5^ as well as orthopaedic pathologies such as bone fragility^6^ and osteoarthritis.^7^

Traditionally it has been difficult to accurately assess the level of human obesity, and estimates of these measures have been proposed via the use of body mass index (BMI) calculations (derived from simple measures of weight/height^2^) and/or simple measures of waist circumference.^8^ However these are prone to error, particularly in tall or short individuals where the variation in height may result in “false negative” or “false positive” identifiers of obesity since the technique does not distinguish between body fat, lean body mass, and other factors such as an individual’s fitness status and cardiovascular risk.^9^ A reliable method for measurement and monitoring of the body adipose tissue distribution is therefore vital in order to better understand and plan clinical interventions to reduce the level of obesity and the burden of associated diseases.

Imaging methods for accurately monitoring changes to human adipose tissue volumes are potentially very useful, since they can provide accurate and non-invasive ways in which response to pharmaceutical or lifestyle interventions can be quantified. Of the numerous imaging modalities available, MRI is particularly useful for this purpose,^10^ since it uses non-ionising radiation which enables repeated use for monitoring over time. The soft-tissue contrast provided by MRI can also highlight the subcutaneous adipose tissue (SCAT) and visceral adipose tissue (VAT) compartments within the abdomen^11^ and these are beginning to form useful imaging biomarkers of abdominal adiposity.

Early studies describing the use of MRI for measurement of abdominal adiposity utilised single axial slices for assessment of fat areas^12^ or a multiple axial slices for assessment of “partial abdomen” fat volumes,^13^ typically using an anatomical locator (*e.g.* specific vertebrae or the umbilicus) for repeatable positioning of the image slices. The current MRI pulse sequence of choice utilises a simple method of fat/water signal separation as originally described by Dixon in 1984.^14^ Over the last few years, this method has been further optimised and combined with improved fast spoiled gradient echo pulse sequences and better gradient magnetic field technology in order to enable the acquisition of extended areas of “whole abdomen” fat volumes from the pelvic floor to the diaphragm.^15^ Various software packages are commercially available for the post-processing analysis of VAT and SCAT and a user comparison of the different packages has previously been reported.^16^

In this pilot study, we sought to address the following: (i) whether the acquisition of “partial abdomen” fat volumes or “whole abdomen” fat volumes provided better correlation with BMI; and, (ii) whether one technique or the other would be better indicated for use in longitudinal studies where small volume changes may be present (*e.g.* following pharmaceutical intervention). For “partial abdomen” acquisitions the post-processing segmentation workload duration is quicker, but the method may be more prone to variations associated with body fat re-distributions between successive scans. Conversely by using the “whole abdomen” method, we hypothesised that scan-to-scan variations might be less, albeit at the expense of extended post-processing segmentation workload duration. The aim of the study therefore was to answer these research questions by scanning a cohort of healthy volunteers on three separate occasions (twice in the supine position and once in the prone position) in order to observe correlations with BMI and “scan-to-scan” variations in measures of VAT and SCAT volumes associated with each acquisition technique.

## METHODS AND MATERIALS

### MRI acquisition

A cohort of fifteen informed and consented healthy volunteers (12 female, 3 male) with mean age 34 years (range 23–50 years) and mean BMI 24.9 kg m^–^^2^ (range 19.4–30.1 kg m^–^^2^) were scanned on a 3T Prisma^Fit^ MRI Scanner (Siemens Healthineers, Erlangen, Germany), using two 18-channel body matrix coils and spine matrix coil to cover the entire abdomen.

For each volunteer, a series of axial 2D dual-echo Dixon Volume Interpolated Breath-hold Examination (VIBE) gradient echo images were acquired through the abdomen from the top of the diaphragm down to the femoral heads in two breath-holds, providing fat-only and water-only images. The imaging parameters were TR 3.67 ms, TE 1.23 and 2.46 ms, flip-angle (FA) 9° and bandwidth 1040 Hz/pixel. Each slice was 3 mm thick, and the number of slices acquired ranged from 144 to 172, depending on the height of the volunteer. The in-plane spatial resolution was 169 × 320 pixels over a typical (patient size dependent) field-of-view (FOV) of 390 × 480 mm. Each breath-hold lasted approximately 10 s.

Three separate acquisitions were acquired, with the volunteer being removed from the scanner and instructed to walk about for a few minutes before being repositioned for each acquisition in order to simulate a completely new scan. Volunteers were scanned twice in the supine position (referred to as “supine 1” and “supine 2”), and once in the prone position (referred to as “prone”). The prone position was used in order to simulate the greatest possible variation in subject positioning. Volunteers were scanned with their arms by their sides when in the supine position, and arms above their head when scanned in the prone position. Padding was used between the abdomen and the arms where required in order to provide clear anatomical separation.

### Image analysis

**Figure 1.**
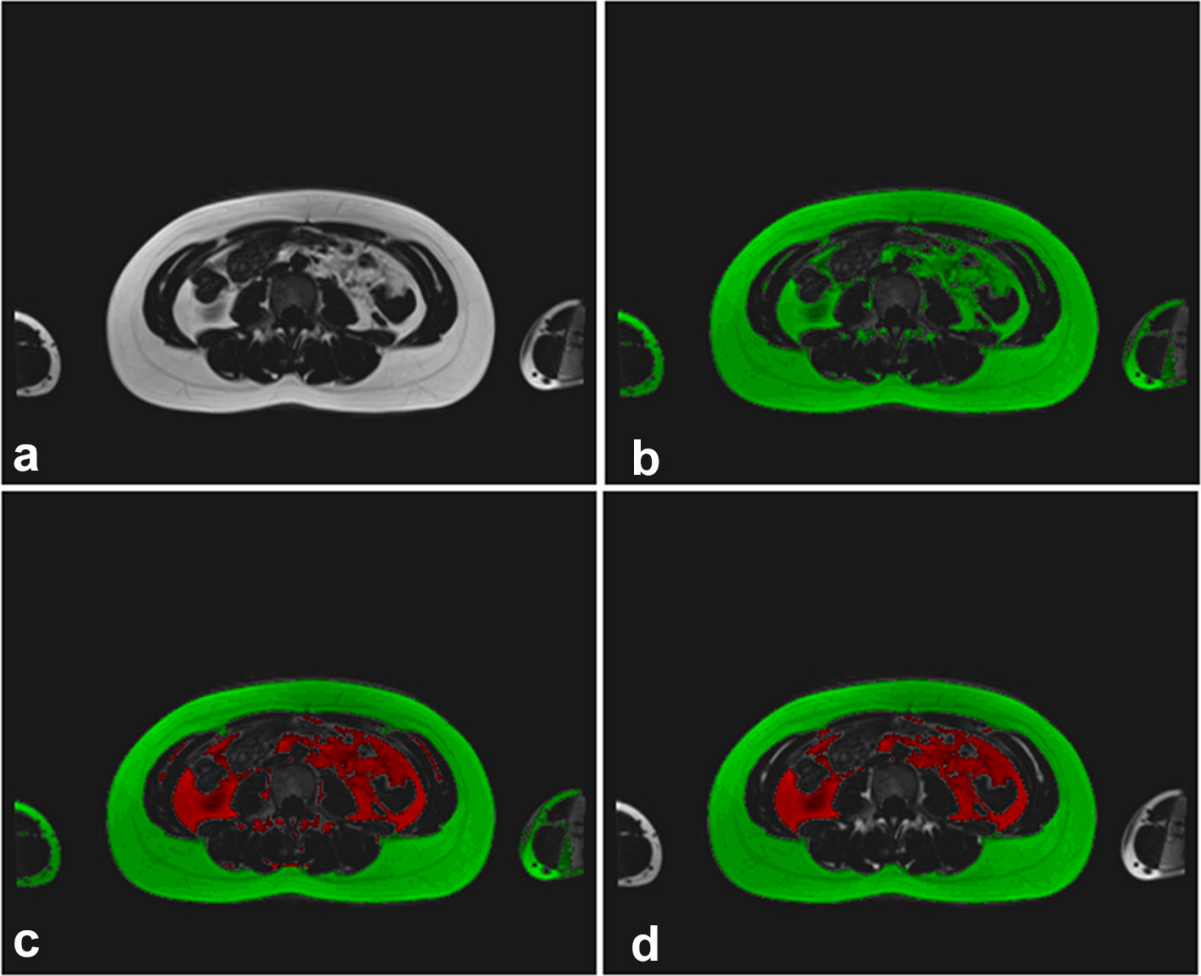
Analysis of images (a) original image, (b) image after global thresholding, (c) separation of visceral (inner) and subcutaneous (outer) adipose tissue applied, (d) final image after analysis complete.

**Figure 2.**
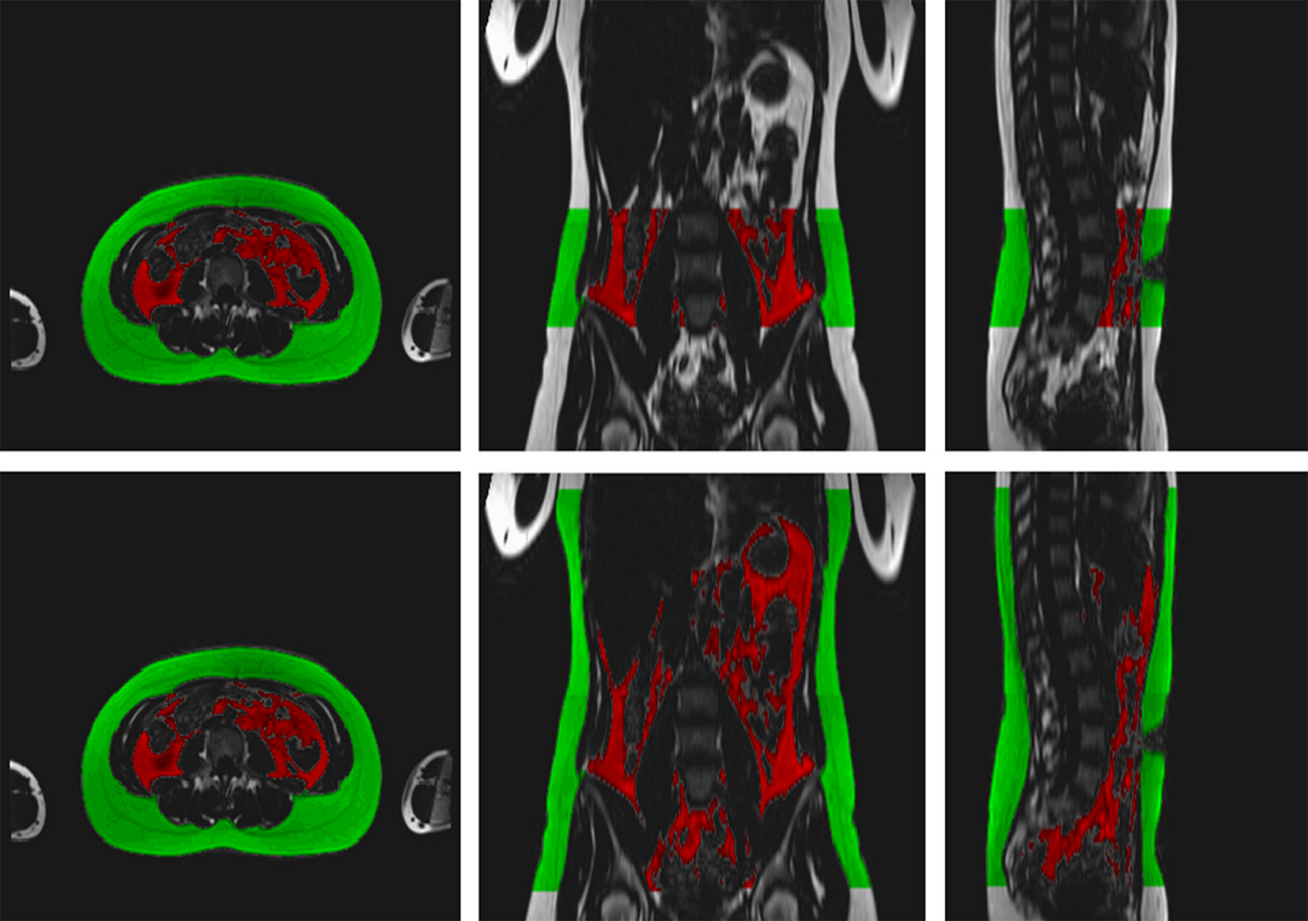
Extent of anatomical regions for partial abdomen coverage, extending from L3–L5 (above) and whole abdomen measurements starting at the dome of the liver and extending down to the top of the fermoral heads (below). Subcutaneous adipose tissue is highlighted (outer) and visceral adipose tissue is highlighted (inner).

Initially, all images were reviewed by an experienced radiologist in order to confirm that no clinical “incidental findings” were present. Following this, the two axial image slice blocks required for each whole volume were combined using ImageJ (U.S. National Institutes of Health, Bethesda, MD) to form a single data set, and any overlapping slices were removed. Image analysis was carried out using Analyze (v. 12.0, Mayo Clinic, Rochester, MN) using the fat-only images. The choice of post-processing package was made on the basis of local availability, together with known suitability for use based on previously performed studies elsewhere.^10, 11,16^ A signal-intensity threshold cut-off value was applied to the images to separate hyper-intense adipose tissue from hypo-intense background and non-adipose tissues. The threshold cut-off value was chosen manually for each data set by an experienced observer. Manual segmentation methods were then used to remove any remaining hyper-intense signal areas (such as bone marrow) that did not correspond to either visceral or subcutaneous adipose tissue, and to correct areas assigned in error by the original signal threshold (Figures 1 and 2).

The dome of the liver (upper) and the top of the femoral heads (lower) were used as anatomical boundaries to determine MRI slice limits for the volume assessments (“whole abdomen”). The segmentation process was then repeated using the top of the L3 intervertebral disc and the base of the L5 intervertebral disc as anatomical boundaries to determine MRI slice limits for the localised volume assessments (“partial abdomen”). The final segmented data sets were used to measure volumes of VAT and SCAT, and the VAT to SCAT ratio. The process was completed for all three acquisitions (“supine 1”, “supine 2” and “prone”).

In order to investigate test–retest intraobserver variation, the “supine 1” volumes were analysed fully for a second time by the same observer after a period of at least 1 month (in order to minimise learning effects). Additionally, the first ten “supine 1” volumes were analysed by a second observer (using the same methodology) to derive a measure of test–retest interobserver variation.

### Statistical analysis

We sought to identify whether patient positioning (“supine 1”, “supine 2” or “prone”) resulted in any statistically significant differences between measured volumes. In order to ascertain this, the measurements of VAT and SCAT for each acquisition were first assessed for normality using the Shapiro-Wilk test. Thereafter, normally distributed data were compared between the three different data acquisitions using the paired *t*-test, and non-normally distributed data were compared using the Wilcoxon signed rank test. The Bonferroni correction was used to account for multiple comparisons. The intraclass correlation coefficient (ICC) was also used to examine correlations between the three different acquisitions for “whole abdomen” and “partial abdomen” VAT and SCAT measurements. The VAT, SCAT and TAT volumes were plotted against volunteer BMI for each patient position to determine which resulted in the best correlation. Bland-Altman analysis was used to highlight individual VAT v SCAT ratio variations between the “partial abdomen” and “whole abdomen” techniques.

Scan-to-scan coefficients of variation (CoV) for “whole abdomen” VAT, SCAT and VAT to SCAT ratios over the three acquisitions (“supine 1”, “supine 2” and “prone”) were calculated for each individual volunteer. These were then combined into a root mean square value (RMS CoV). This was repeated for the “partial abdomen” measurements, and the RMS CoV’s were compared to determine which coverage method provided the better scan-to-scan reproducibility. The process was also repeated after omitting the “prone” volumes, as this was considered to be an extreme change in patient positioning (*i.e.* unlikely to be reflective of clinical practice). Finally, RMS CoV’s for the original measurement of “supine 1” and the repeated measurements were also calculated to quantify test–retest intra- and interobserver variations. All statistical tests were performed using SPSS (v. 22.0, IBM Corp, Armonk, NY) and *p*-values of less than 0.05 were considered to be statistically significant.

## RESULTS

All volunteers completed the study successfully, resulting in 45 “whole abdomen” measurements and 45 “partial abdomen” measurements being used for scan-to-scan statistical comparisons.

The measured mean volumes (± SD) for SCAT, VAT and VAT to SCAT ratio are shown in Table 1. When the data were tested for normality using the Shapiro Wilk test, the SCAT volumes were found to be normally distributed (enabling parametric test comparison of means) whilst the VAT volumes were non-normally distributed (requiring non-parametric test comparison of means). For both the “whole abdomen” and “partial abdomen” volumes, no significant differences were noted between the means of the VAT volumes or the calculated ratios. However the mean SCAT volumes measured in the prone position were each found to be significantly lower than those measured in the supine position (*p* < 0.001). All ICC comparisons were categorised as “excellent”, and ranged from 0.97 (“supine 1” v “prone” for VAT “partial abdomen”) to 0.99 (“supine 2” v “prone” for VAT “whole abdomen”).

**Table 1.** Average measured VAT and SCAT volumes (measured in litres, L) and VAT to SCAT ratios for the 3 acquisitions

		VAT (L)	SCAT (L)	VAT:SCAT ratio
Whole abdomen volumes	Supine 1	2.42 ± 2.12	7.58 ± 2.11	0.35 ± 0.38
Supine 2	2.39 ± 2.05	7.55 ± 2.13	0.34 ± 0.38
Prone	2.40 ± 2.07	7.18 ± 1.99	0.37 ± 0.42
Partial abdomen volumes	Supine 1	0.82 ± 0.72	2.64 ± 0.85	0.35 ± 0.42
Supine 2	0.79 ± 0.67	2.69 ± 0.88	0.34 ± 0.42
Prone	0.76 ± 0.60	2.49 ± 0.81	0.36 ± 0.43

SCAT, subcutaneous adipose tissue; VAT, visceral adipose tissue.

**Figure 3.**
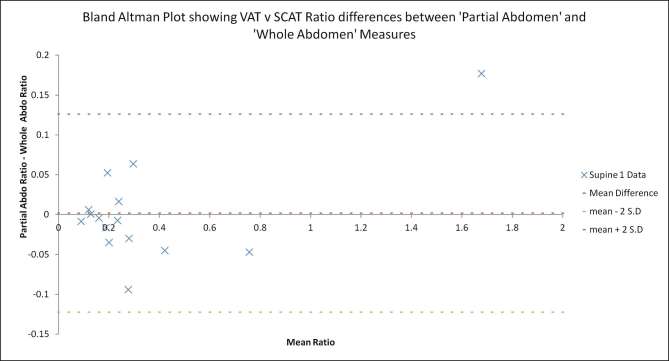
Bland Altman plot of differences between VAT to SCAT ratios from “partial abdomen” and “whole abdomen” measures. SCAT, subcutaneous adipose tissue; VAT, visceral adipose tissue.

For the VAT to SCAT ratio (a measure often used clinically), the mean values of the “partial abdomen” ratios were noted to be similar to the mean “whole abdomen” ratio measurements overall (range 0.34–0.37—Table 1). However when the data were examined on an individual basis, there were some notable differences evident (Figure 3). Figure 3 shows that the VAT to SCAT ratio differences for “supine 1” measurements were as high as 0.17 (“partial abdomen” ratio proportionally higher) and as low as −0.09 (“partial abdomen” ratio proportionally lower). Although not plotted, the equivalent data for the “supine 2” and “prone” acquisitions were very similar.

Table 2 consists of *r*^2^ values describing correlations between VAT, SCAT and TAT with BMI for each volunteer. All measures showed a positive correlation with BMI. The TAT measurement was strongly correlated with BMI in all instances, but measures of VAT in particular were only weakly correlated with BMI. In all cases the “whole abdomen” measures were more strongly correlated with BMI than the “partial abdomen” measurements.

**Table 2.** *r*^*2*^ values for VAT, SCAT and TAT measurements plotted against volunteer BMI

		VAT	SCAT	TAT
Whole abdomen volumes	Supine 1	0.33	0.40	0.74
Supine 2	0.32	0.43	0.74
Prone	0.30	0.38	0.71
Partial coverage volumes	Supine 1	0.29	0.30	0.65
Supine 2	0.30	0.30	0.66
Prone	0.25	0.30	0.62

SCAT, subcutaneous adipose tissue; TAT, total adipose tissue; VAT, visceral adipose tissue.

All assessments of scan-to-scan variation, along with intra- and interobserver variation are included in Table 3. For the assessment of scan-to-scan variation, the “whole abdomen” analyses for VAT and SCAT (4.16 and 3.61%) were less variable than those for the “partial abdomen” measurements (6.31 and 5.07%) respectively.

**Table 3.** RMS coefficients of variation for different reproducibility measures of VAT and SCAT

	Reproducibility measure	VAT RMS CoV (%)	SCAT RMS CoV (%)
Whole abdomen volumes	Scan-to scan	4.16	3.61
Supine only scan-to scan	2.66	1.34
Intraobserver	3.50	0.97
Inter-observer	6.78	2.93
Partial coverage volumes	Scan-to scan	6.31	5.07
Supine only scan-to scan	4.80	3.13
Intraobserver	3.47	1.17
Interobserver	6.77	2.14

RMS CoV, root-mean-square coefficients of variation; SCAT, subcutaneous adipose tissue; VAT, visceral adipose tissue.

When the “prone” volumes were removed, the RMS CoV’s for both measures were reduced further. The RMS CoV’s for the “whole abdomen” assessments VAT and SCAT were 2.66 and 1.34% respectively, compared to 4.80 and 3.13% for the “partial abdomen” measurements VAT and SCAT. Again whole abdomen analyses were less variable.

For all single time-point test–retest measurements there was little difference in repeatability between “whole abdomen” and “partial abdomen” volumes. However, the intraobserver RMS CoV’s for VAT and SCAT were much lower than the equivalent interobserver RMS CoV’s. Intraobserver variations were recorded as low as 0.97% (for “whole abdomen” SCAT) whilst interobserver variations were recorded as high as 6.78% (for “whole abdomen” VAT). Scan-to-scan variations were slightly larger than test retest intraobserver variations, but smaller than test retest interobserver variations—suggesting that for small clinical studies where volume changes are examined over time it would be preferable to have a single observer carry out the analysis. In all cases, the VAT measurements were more variable than the SCAT measurements, reflecting the difficulty associated with measuring the former variable.

## DISCUSSION

In this pilot study, we have demonstrated that the implementation of MRI segmentation techniques using commercially available software can provide reproducible quantitative measures of “whole abdomen” volumes (from commercial Dixon sequences) that are potentially suitable for use in longitudinal MR abdominal adiposity studies. When compared with “partial abdomen” techniques, the “whole abdomen” volume measurements (TAT and SCAT) were found to correlate more strongly with BMI, although the measurement of visceral adipose tissue (VAT) remained poorly correlated with BMI.

This research supports the theory that BMI alone may not be a reliable indicator of overall metabolic health,^17^ as it correlates very weakly with VAT measurements despite its strong correlation with TAT. It is widely accepted that the measure of BMI cannot distinguish between lean body mass and fat body mass. Further, there is good evidence to suggest that VAT is a particularly important clinical measure since it is known to correlate more strongly (relative to SCAT) with adverse metabolic risk indices such as measured cholesterol and blood pressure, but more weakly (relative to SCAT) with BMI.^18^ High levels of VAT are more associated with a range of clinical conditions than TAT or SCAT.^19^ Although not directly applicable to our cohort of healthy volunteers in this study, the concept of the “obesity paradox”^20^ further suggests that the measurement of BMI in patients with cardiovascular disease may not accurately predict the status of a better or worse clinical outcome. Whether more quantitative measures such as VAT are better able to predict clinical outcome remains to be seen, but this particular research question is an intriguing one.

In this study, the DIXON method used for anatomical coverage of the full abdominal cavity shares “outline similarity” with work reported elsewhere^21^–albeit with different segmentation approaches. The reproducibility of our work is also similar to that published recently by Middleton et al.^22^ From a methodological perspective, our original hypothesis was based on the fact that whole abdomen volumes would provide a more reproducible MR endpoint relative to the commonly used partial coverage approach. To a large extent this has been confirmed, where for example “whole abdomen” measurements demonstrated less variation in all scan-to-scan examinations than the equivalent “partial abdomen ” measurements. The lower variation in “whole abdomen” measurements indicates that these would be well suited for longitudinal research studies, especially if only small changes are expected. However, the interobserver variation was typically twice as large as the intraobserver variation - implying that the same observer should make measurements throughout such a study where possible.

In this work, the VAT and SCAT volumes themselves were not corrected for possible variations associated with magnetic field inhomogeneities at the edges of the FOV. This distortion effect can occur due to gradient non-linearities away from the scanner isocentre. In order to minimise this effect we performed the following: (i) we acquired all images with the scanner vendor “distortion correction” algorithm applied; (ii) we ensured that all scanning was performed with the centre of the FOV “at isocentre”, and (iii) we ensured that the two overlapping FOV’s were acquired such that the anatomical area of interest lay as close to the isocentre as possible in the z-direction—*i.e.* outer areas of the FOV in the z-direction were discarded.

In order to explore the widest range of scan-to-scan conditions, we elected to scan the same volunteers on three different occasions—twice in the “supine” position and once in the “prone” position. Although there were no statistically significant differences between any of the measurement means between the two supine volumes, the mean SCAT volumes were consistently and significantly lower when measured prone. Changing the patient from “supine” to “prone” was implemented in order to represent the maximum possible amount of radiographic variation in patient position and therefore the highest chance of redistribution of adipose tissue that might be expected. It is possible that when the patients positioned their arms above their head in the prone position they “stretched out”, thus resulting in a redistribution of a portion of the SCAT volume outside of the measurement field. This effect is marginally more pronounced for the “partial coverage” measurements since the redistribution of the adipose tissue volume can occur more easily above and below the plane of the image slices under investigation.

In this study, it was interesting to note that the mean VAT to SCAT volume ratio remained relatively consistent, whether derived from “partial abdomen” or “whole abdomen” measures. This implies that for a single time-point clinical overview the “partial abdomen” measurements may be sufficient as a way of deriving this particular variable. However detailed inspection of individual results (Figure 2) revealed large “per-volunteer” differences between the VAT to SCAT volume ratios when calculated using either “whole abdomen” or “partial abdomen” data—suggesting that the consistency between the means may be a chance observation. This is accepted as a possible weakness of our study; an investigation involving a greater number of volunteers may help to confirm whether this consistency between the VAT to SCAT volume ratios is real or a statistical artefact.

Other weaknesses of our study include the relatively large interobserver variation—believed to be attributable in part to the choice of signal intensity “cut-off” threshold used to differentiate adipose tissue from other structures. This threshold signal intensity was chosen manually by each observer independently, and small variations to the chosen threshold may potentially contribute to a large variation in the measured volumes. This is particularly likely to be the case for VAT, since it is a more heterogeneous structure and also closely associated alongside other tissue structures with variable signal intensity values. In this study, the VAT measurements were found to be consistently less reproducible than the SCAT measurements. The final weakness of the work was the time required to perform “whole abdomen” segmentation (typically 30–60 min of processing time per data set). This could potentially preclude the use of the segmentation technique for large-scale population studies; although automated methods^23^ for this processing are evolving any may reach commercial platforms for wider use at some stage in the future.

In conclusion, we have reported a commercially available 3T MRI method that is able to acquire and measure “whole abdomen” adipose tissue volumes. In a cohort of healthy volunteers, the “whole abdomen” volumes were better correlated with BMI than commonly used “partial abdomen” measures, and the “whole abdomen” technique was more reproducible when measured over multiple time-points. These variables are deemed suitable for use as clinical MRI biomarkers in longitudinal radiology studies where small compartmental changes to abdominal adipose tissue volumes may be expected.
